# Wood degradation by *Fomitiporia mediterranea* M. Fischer: Physiologic, metabolomic and proteomic approaches

**DOI:** 10.3389/fpls.2022.988709

**Published:** 2022-09-26

**Authors:** Marion Schilling, Alessandra Maia-Grondard, Raymonde Baltenweck, Emilie Robert, Philippe Hugueney, Christophe Bertsch, Sibylle Farine, Eric Gelhaye

**Affiliations:** ^1^Université de Lorraine, INRAE, IAM, Nancy, France; ^2^Université de Strasbourg, INRAE, SVQV UMR-A 1131, Colmar, France; ^3^Université de Lorraine, INRAE, A2F, Nancy, France; ^4^Laboratoire Vigne Biotechnologies et Environnement UPR-3991, Université de Haute Alsace, Colmar, France

**Keywords:** Esca, white rot, *Fomitiporia mediterranea*, grapevine wood, adaptation

## Abstract

*Fomitiporia mediterranea* (Fmed) is one of the main fungal species found in grapevine wood rot, also called “amadou,” one of the most typical symptoms of grapevine trunk disease Esca. This fungus is functionally classified as a white-rot, able to degrade all wood structure polymers, i.e., hemicelluloses, cellulose, and the most recalcitrant component, lignin. Specific enzymes are secreted by the fungus to degrade those components, namely carbohydrate active enzymes for hemicelluloses and cellulose, which can be highly specific for given polysaccharide, and peroxidases, which enable white-rot to degrade lignin, with specificities relating to lignin composition as well. Furthermore, besides polymers, a highly diverse set of metabolites often associated with antifungal activities is found in wood, this set differing among the various wood species. Wood decayers possess the ability to detoxify these specific extractives and this ability could reflect the adaptation of these fungi to their specific environment. The aim of this study is to better understand the molecular mechanisms used by Fmed to degrade wood structure, and in particular its potential adaptation to grapevine wood. To do so, Fmed was cultivated on sawdust from different origins: grapevine, beech, and spruce. Carbon mineralization rate, mass loss, wood structure polymers contents, targeted metabolites (extractives) and secreted proteins were measured. We used the well-known white-rot model *Trametes versicolor* for comparison. Whereas no significant degradation was observed with spruce, a higher mass loss was measured on Fmed grapevine culture compared to beech culture. Moreover, on both substrates, a simultaneous degradation pattern was demonstrated, and proteomic analysis identified a relative overproduction of oxidoreductases involved in lignin and extractive degradation on grapevine cultures, and only few differences in carbohydrate active enzymes. These results could explain at least partially the adaptation of Fmed to grapevine wood structural composition compared to other wood species, and suggest that other biotic and abiotic factors should be considered to fully understand the potential adaptation of Fmed to its ecological niche. Proteomics data are available *via* ProteomeXchange with identifier PXD036889.

## Introduction

Grapevine, *Vitis vinifera* L., is one of the most economically important cultures in the world: it is produced worldwide and its production value was estimated to more than $73billions in 2018 (Food and Agriculture Organization of the United Nations Statistics (FAOSTATS), 2021). This high valued production is however threatened by many vineyard diseases. Among them, Grapevine Trunk Diseases (GTDs) are considered as the most challenging one (Reis et al., 2019). In 2012 and 2013, economic losses of, respectively, 11 and 13% of the French vineyard were attributed to GTDs (Doublet et al., 2014). No satisfying treatment exists yet, even if promising solutions developments are ongoing (Gramaje et al., 2018; Mondello et al., 2018).

Esca is one of the main GTDs, causing alone 3% of French vineyard economical losses in 2013 (Doublet et al., 2014). Its importance tends to increase over the last years in France and other European countries (Bruez et al., 2013; Guerin-Dubrana et al., 2019). Esca affects mainly mature vines and is considered as a complex of diseases, associated with various fungal species and symptoms (Mugnai et al., 1999; Surico et al., 2008; Lecomte et al., 2012; Bruez et al., 2014). Esca symptoms are usually grouped into five syndromes described as: (i) brown wood streaking, affecting rooted cuttings and, associated with internal brown wood necrosis, (ii) Petri disease, affecting young vines and associated with wood and crown symptoms but also foliar symptoms such as leaf chlorosis and yield or vigor loss, (iii) Young Esca or Grapevine Leaf Stripe Disease (GLSD), associated with wood and crown symptoms as well as foliar symptoms such as the typical “tiger-striped” leaves or even apoplexy of the vine, (iv) Esca, affecting mainly mature vines, characterized by typical white-rot in wood and also called “amadou,” and (v) Esca proper, a combination of GLSD and Esca syndromes (Surico, 2009; Bertsch et al., 2013). Among the GTD-associated fungi, *Phaeomoniella chlamydospora* and *Phaeoacremonium minimum* were the main identified species in wood necrosis observed in the first three described syndromes (brown wood streaking, Petri disease and young Esca; Surico, 2009). In wood affected by Petri disease, *P. chlamydospora* is the main identified fungal species but up to 36 other species were also isolated, including *P. minimum* and *Cadophora luteo-olivacea* as the most prevalent (Pilar Martínez-Diz et al., 2021). Other species not directly associated with Esca such as *Eutypa lata* or *Pleurostomophora richardsiae* could also be present in wood necrosis, and the role of each associated fungus is still not fully understood (White et al., 2011; Canale et al., 2019). In white-rot associated with Esca and Esca proper syndromes, the main identified fungi are Basidiomycota and the main genus is *Fomitiporia*. In Europe, the most represented species is *Fomitiporia mediterranea* (Fmed) M. Fischer (Hymenochaetaceae, Hymenochaetales, Basidiomycota; Fischer, 2002; Surico et al., 2008; Bruez et al., 2020; Moretti et al., 2021).

Grapevine trunk diseases (GTDs)-associated fungi colonize grapevine wood but not the leaves. Their role in foliar symptoms still need further research (Fontaine et al., 2016; Del Frari et al., 2021). More particularly for Fmed, evidence for its pathogenicity *stricto sensu* is still lacking. *In vitro*, Fmed indeed induces necrosis on detached grapevine wood (Markakis et al., 2017) and inhibits growth of grapevine protoblasts (Bruno and Sparapano, 2006a). After inoculation, Fmed is able to induce white-rot only on mature vines, but without typical Esca foliar symptoms (Moretti et al., 2021). Still, curettage of Esca-affected vines which consists of removing white-rot zone, where Fmed is found in majority, improves vine resilience and reduces Esca symptoms the following years (Cholet et al., 2021; Pacetti et al., 2021). Moreover, Fmed was found in higher abundancy in wood showing foliar symptoms (Del Frari et al., 2021). One hypothesis of these observations is that Esca leaf symptoms might require the presence in wood of both Fmed, or other *Hymenochaetales*, and tracheomycotic fungi (Del Frari et al., 2021).

Fmed could be functionally classified as a white-rot fungus (WRF), as it is able to mineralize all wood structure components, including lignin, the most recalcitrant one (Floudas et al., 2012; Sahu et al., 2021). Due to grapevine wood specificities, Fmed might be specifically adapted to grow on this wood species (Schilling et al., 2021). As with other WRF, genomic studies revealed that Fmed has a large variety of enzymes which could be involved in the extracellular degradation of lignin, and in particular class II peroxidases (Class-II PODs) and laccases. Class-II PODs are classified upon their structure and function as Manganese Peroxidases (MnPs), Lignin Peroxidases (LiPs), Versatile Peroxidases (VPs) and Generic Peroxidases (GPs). The Fmed genome harbors 16 gene encoding MnPs (including 3 short MnPs, 11 long MnPs and 2 atypical MnPs), and 1 gene encoding a GP (Floudas et al., 2012). In contrast with other fungi such as *Trametes versicolor* (Tver; L.) Lloyd, 1921 (Polyporaceae, Polyporales, and Basidiomycota), a WRF found on a large range of forest hardwoods and some softwoods, no genes encoding LiPs or VPs were found. LiPs cleave lignin ß-1 linkage (non-aromatic part) at their typical catalytic tryptophan site, by oxidation and in the presence of hydrogen peroxide as electron acceptor (Falade et al., 2017), while MnPs possess a manganese (II) oxidation site and use chelated manganese (III) ions as electron donor, to oxidize lignin phenolic structures (Hofrichter, 2002). Concerning VPs, they exhibit both a catalytic tryptophan site, like LiPs, and a manganese (II) oxidation site, like MnPs, whereas GPs show none of them. The latters probably act by oxidizing their substrate at their main heme channel (Floudas et al., 2012). The functional differences between LiPs and MnPs were suggested to be involved in specific WRF lignin degradation capacity, depending on lignin composition (Suzuki et al., 2012). Two main degradation patterns were described for WRF: (i) simultaneous (or nonselective), when lignin, cellulose and hemicelluloses are simultaneously degraded, or (ii) selective (or preferential), when lignin is preferentially degraded (Blanchette, 1991). However, it was not shown if LiPs or MnPs could favor one of the two patterns. For example, two WRF, Tver and *Phanerochaete chrysosporium* strain BKM F-1767, both possessing LiPs and MnPs, were shown to cause, respectively, simultaneous and selective degradation (Blanchette et al., 1989).

Laccases are multicopper oxidases acting on a large range of substrates, including lignin and lignin residues, reducing O_2_ to H_2_O (Reiss et al., 2013; Manavalan et al., 2015). However, their role in lignin degradation remains unclear. These enzymes are able to oxidize phenolic compounds suggesting also a role in detoxification of potential toxic compounds found in wood and in particular in grapevine wood. Fmed genome analysis revealed the presence of ten genes encoding laccases (*sensu stricto*) and one Fet3 ferroxidase (Floudas et al., 2012). Recent investigation of laccases and MnPs activities in Fmed culture media confirmed their importance for Fmed growth on wood (Pacetti et al., 2022).

Enzymes involved in polysaccharide degradation, in particular hemicelluloses and cellulose, belong to Carbohydrate-Active enZYmes (CAZYs). They can be highly specific relative to sugar units or linkage types (King et al., 2011; Hage and Rosso, 2021). Among them, Glycoside Hydrolases (GHs) catalyze polysaccharide hydrolysis with high carbohydrate and sugar conformation specificities (Coines et al., 2019). Depending on their molecular mechanisms they are classified into 173 families (AFMB, 2022b). The Fmed genome exhibits 50 GHs from nine families. Carbohydrate Esterases (CEs) catalyze O- or N-deacetylation of heteropolysaccharides, i.e., hemicelluloses and pectins, and are further classified into 20 families (AFMB, 2022a). They are key enzymes to enable further polysaccharide depolymerization by GHs. Twelve genes encoding CEs from four families were detected in the Fmed genome (Floudas et al., 2012). Auxiliary activity enzymes are redox enzymes acting conjointly with CAZYs. Firstly classified as GH61 family, Lytic Polysaccharides Mono-Oxygenases (LPMOs) are now classified as Auxiliary Activity enzymes family 9 (AA9), since evidence for their oxidative activity involved in polysaccharides degradation was obtained (Rani Singhania et al., 2021). Thirteen genes encoding LPMOs were identified in the Fmed genome (Floudas et al., 2012).

Wood not only contains structural components, but also extractives, which are small molecules or metabolites easily extractible with solvent, often associated with antimicrobial or antifungal properties and involved in wood durability (Valette et al., 2017; Perrot et al., 2020). Grapevine wood extractives belong mainly to the stilbene family or to other phenolics such as flavonoids or phenolic acids. Many of them are known for their antifungal and antioxidant activities (Jeandet et al., 2002). Antifungal activities against Fmed were demonstrated *in vitro* for six stilbenes (resveratrol, pterostilbene, miyabenol C, isohopeaphenol, and vitisin A and B; Bruno and Sparapano, 2006b; Lambert, 2011; Lambert et al., 2012). However, as shown with other wood-decaying fungi, Fmed has developed both intracellular and extracellular detoxification processes to deal with such extractives. Intracellular detoxification by wood-decaying fungi involve enzymes constituting the fungal xenome, which is mainly represented by cytochrome P450s and glutathione transferases (Morel et al., 2013; Morel-Rouhier, 2021). The Fmed genomes showed, respectively, 130 and 38 genes encoding for those two enzymes families (Floudas et al., 2012; Morel et al., 2013). In parallel, Fmed extracellular detoxification was suggested, but mechanisms remain not fully understood. It was shown that the presence of resveratrol in Fmed cultures induced laccase activity and a decrease of resveratrol concentration, concomitantly to the production of two non-identified by-products (Bruno and Sparapano, 2006b). Moreover, a 60 kDa laccase isolated from Fmed secretome was shown to oxidize resveratrol, and four non-identified products (mass to charge ratios (m/z) of 471 [M–H]^−^ and 499 [M–H]^−^ for two of them) were detected by mass spectrometry (Abou-Mansour et al., 2009). Oxidation and polymerization of resveratrol into stilbenes dimers by *Botrytis cinerea* Pers., an Ascomycota and grapevine fruits pathogen, have been described (Cichewicz et al., 2000). Here should be noticed that stilbenes oligomers are usually associated with antifungal activities (Lambert et al., 2012; Stempien et al., 2017). Thus, dimerization was suggested to be a first step in fungal resveratrol metabolization (Cichewicz et al., 2000).

In this study, we aimed to investigate whether or not Fmed is better adapted to degrade grapevine wood compared to other wood species, possibly due to specific degradation or detoxification mechanisms. Tver, well studied for fungal wood degradation mechanisms, was used as a model. CO_2_ production rate was measured during 3-month cultures of Fmed and Tver on grapevine cv. “Gewurztraminer,” beech (*Fagus sylvatica*) or spruce (*Picea abies*) wood sawdust, to follow carbon mineralization continuously. Global mass loss and wood fiber chemistry (cellulose, hemicelluloses, lignin and soluble compounds contents), as well as targeted metabolomic (for primary metabolites and extractives) analysis were conducted to compare wood degradation upon substrates for the two WRF, and proteomic analysis at 1 and 3 months of culture aimed to correlate those observations with the fungal secretome. This constitutes a first step for a comprehensive approach of Fmed colonization mechanisms on wood.

## Materials and methods

### Fungal strains

*Fomitiporia mediterranea* (Fmed) strain phco36 was provided by Laboratoire Vigne Biotechnologies et Environnement (LVBE) of University of Haute-Alsace in Colmar and isolated from *Vitis vinifera* L. cv. ‘Ugni blanc’ in 1996 in Saint-Preuil (Charente, France). *Trametes versicolor* (Tver) strain BRFM1218 was obtained from the Center International de Ressources Microbiennes (CIRM) catalog and isolated from *Quercus* sp. wood in France. All strains were cultivated and regularly regenerated on modified Pachlewski solid culture media (P25), composed of 20 g/l agar, 20 g/l glucose, 1.0 g/l potassium phosphate (KH_2_PO_4_), 0.5 g/l magnesium sulfate (MgSO_4_), 3.0 g/l ammonium tartrate (C_4_H_4_O_6,_2(NH_3_)), 5.0 g/l maltose, 0.4 g/l thiamine, 10 mg/l MnCl_2_, 12 mg/l FeSO_4_, 7H_2_O, 4.6 mg/l ZnSO_4_, 7H_2_O, 1.2 mg/l CuCl_2_, 17 mg/l H_3_BO_3_, and 1.4 mg/l NaMoO_4_, 2H_2_O.

### Wood material

Mature trunks of *Vitis vinifera* cv. “Gewurztraminer” were cut in the Soultzmatt vineyard (Haut-Rhin, France) in February 2021 with the kind agreement of Valentin Zusslin Estate (Orschwihr, Haut-Rhin, France), and stored at room temperature before being sawn. Only white parts of the vine trunks, considered as healthy, were selected, sawn into 1 cm^3^ pieces and further reduced into sawdust by liquid-nitrogen assisted cold ball grinding (Cryomill^®^. Retsch GmBH). Bark, necrosis and black or brown parts were discarded during sawing. Beech (*Fagus sylvatica*) wood sawdust was provided by the BEF laboratory (Biogéochimie des Ecosystèmes Forestiers, INRAE) from Champenoux forest (Meurthe-et-Moselle, France). Spruce (*Picea abies* Karst) wood sawdust was provided by the NIBIO laboratory (Norwegian Institute of Bioeconomy Research) from Norwegian forest stands. All sawdusts were sifted between 0.5 and 2 mm and dried at 105°C before inoculations.

### Fungal cultures on sawdust

**Figure 1:**
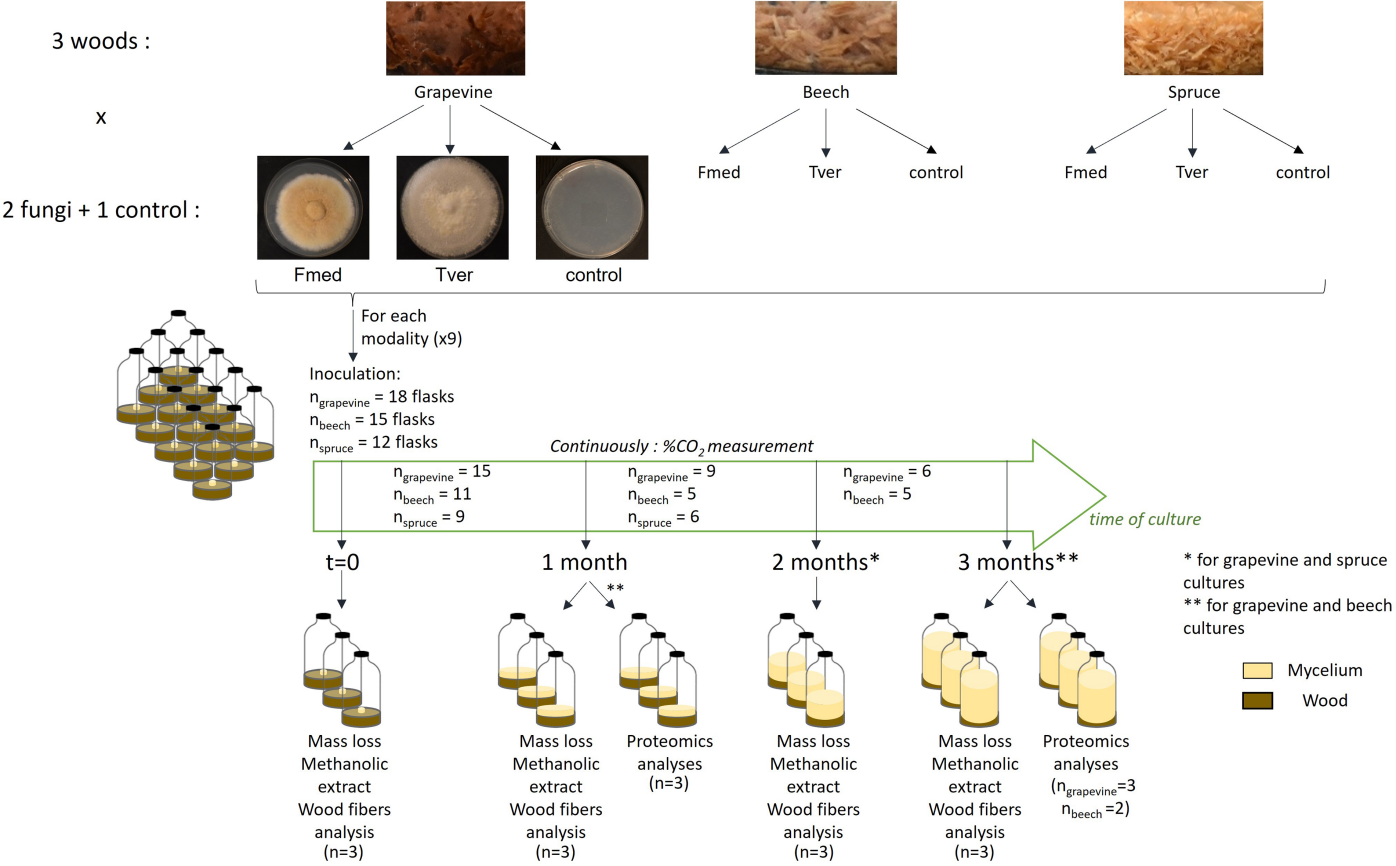
Experimental design.

The experimental plan is summarized in Figure 1. Wood sawdust was dried for 48 h at 105°C and 2.0 g per individual were added in 120 ml glass flasks and sterilized for 20 min at 120°C. To rich substrate moisture saturation, 5.0 ml of liquid modified P25 culture media, without carbon source (1.0 g/l KH_2_PO_4_, 0.5 g/l MgSO_4_, 2.2 g/l) ammonium sulfate [SO_4_2(NH_4_), 0.4 g/l thiamine, 10 mg/l MnCl_2_, 12 mg/l FeSO_4_, 7H_2_O, 4.6 mg/l ZnSO_4_, 7H_2_O, 1.2 mg/l CuCl_2_, 17 mg/l H_3_BO_3_, and 1.4 mg/l NaMoO_4_, 2H_2_O], were added under sterile conditions. One cm^3^ inoculum from 2-week-old fungal cultures grown on solid P25 media at 27°C was added to each flask. For control flasks, a similar amount of solid P25 media was added. Within 2 h after inoculation, three flasks of each test combination (9 combinations of wood (× 3) and fungi (× 2) or control, see Figure 1), further identified as time 0, were freeze–dried, lyophilized and ground into powder by liquid-nitrogen assisted cold ball grinding (Cryomill^®^ Retsch GmBH) for methanolic extraction and wood fibers analyses. All other inoculated flasks were closed hermetically with septum caps and stored in the dark at 27°C until sampling (Figure 1). After 1, 2, and/or 3 months of culture (Figure 1), three flasks per test were freeze-dried and lyophilized for mass loss and ground into powder for methanolic extractions and wood fibers analyses. At 1 and 3 months for grapevine and beech sawdust cultures, three other flasks per test were extracted for proteomic analysis.

### Measurement of carbon mineralization

Carbon mineralization was calculated by measuring the CO_2_ pressure in each flask every 1–4 days. CO_2_ content in each flask was measured in %*v*/*v* (%CO_2_) with a Lambda CZ s.r.o. carbometer by flushing air contained in the flask with a KNF Laboport^®^ N86 kt.18 pump and sterile needles connected to silicone pipes through 0.2 μm filter. Oxygen pressure was renewed after each measurement at atmospheric pressure. %CO_2_ was converted into CO_2_ amount (nCO_2_) in mol/L according to ideal gas law:


$$n\mathrm{CO}2=\frac{P\left(\mathrm{CO}2\right)*V}{R*T}$$

where P (CO2) = %CO_2_ multiplied by atmospheric pressure (101,325 Pa)_,_ *V* = 150 ml corresponding to the flask (120 ml) and pipes (30 ml) volume, *R* = 8,314 J mol^−1^ K^−1^ and *T* the temperature of the room in Kelvin during measurement. Finally, nCO_2_ was converted in mass of mineralized carbon (mg C) in mg and the mineralization rate was calculated in mg C/day at each measurement point. Maximal mineralization rate corresponds to the mean of all highest mineralization rates (i.e., all rates which were not significantly different to the highest mineralization rate, according to Tukey test).

### Mass loss

Global mass loss per flask was measured the difference between cultures dry weight after lyophilization and control mean dry weight and is expressed in percentages of control mean dry weight. Control samples dry weight at initial time and after each month of culture showed no difference. Statistical analysis was performed both between each mass loss and its respective control, and between each relevant mass losses with Student–Newman–Keuls test.

### Wood fibers analyses

Wood fibers analyses were performed by ASIA platform (Université de Lorraine-INRAE; https://a2f.univ-lorraine.fr/en/asia-2/). Lignin, cellulose, hemicelluloses and soluble compounds contents were quantified in lyophilized ground samples using a protocol adapted from Van Soest and McQueen (1973). 0.4 g of each sample were dried at 105°C in porous and resistant bags (Fibrebag^®^, Gerhardt GmBH) and submitted to three successive baths to dissolve, respectively, soluble compounds, hemicelluloses and finally cellulose. Baths consisted, respectively, of (i) heated neutral detergent solution composed of: 30 g/l sodium dodecyl sulfate, 18.6 g/l sodium ethylene diamine tetra-acetate, 4.56 g/l sodium phosphate monobasic, 6.81 g/l sodium tetraborate decahydrate 10 ml/l and triethylene glycol, (ii) heated acidic detergent solution with 20 g/l of cetyltrimethylammonium bromure in 0.5 M sulfuric acid, and (iii) 72% sulfuric acid solution at room temperature. Samples dry weights before and after each bath were used to obtain each wood structure compound contents.

### Methanolic extraction

As internal standard, 5 mg ± 0.1 mg of lyophilized and powdered sample were extracted with 250 μl of LC–MS grade methanol previously supplemented with 5 μg/ml chloramphenicol (Sigma-Aldrich). The samples were sonicated in an ultrasonic bath (FB15050, Fisher Scientific, Hampton, United States) for 10 min and centrifugated at 12,000 *g* for 15 min. The supernatant was analyzed using by ultra-high-performance liquid chromatography coupled to high resolution mass spectrometry (UHPLC–HRMS).

### Targeted metabolomic analysis

Metabolomic analyses were performed using a Dionex Ultimate 3,000 UHPLC system (Thermo Fisher Scientific, Waltham, MA, United States). Liquid chromatography-mass spectrometry (LC–MS) grade methanol and acetonitrile were purchased from Roth Sochiel (France); water was provided by a Millipore water purification system. The chromatographic separations were performed on a Nucleodur C18 HTec column (150 mm × 2 mm, 1.8-μm particle size; Macherey-Nagel, Düren, Germany) maintained at 30°C. The mobile phase consisted of acetonitrile/formic acid (0.1%, *v*/*v*, eluant A) and water/formic acid (0.1%, *v*/*v*, eluant B) at a flow rate of 0.3 ml/min. The gradient elution was programmed as follows: 0–1 min, 95% B; 1–6 min,95–0% B; 6–8 min, 0% B. The sample volume injected was 1 μl. The UHPLC system was coupled to an Exactive Orbitrap mass spectrometer (Thermo Fisher Scientific), equipped with an electrospray ionization (ESI) source operating in positive and negative mode. Parameters were set at 350°C for the ion transfer capillary temperature and 2,500 V for the needle voltages. Nebulization with nitrogen sheath gas and auxiliary gas was maintained at 40 and 5 arbitrary units, respectively. The spectra were acquired within the mass-to-charge ratio (m/z) ranging from 75 to 1,500 atomic mass unit, using a resolution of 50,000 at m/z 200 atomic mass unit. The system was calibrated internally in positive mode using dibutyl-phthalate as the lock mass at m/z 279.1591, giving a mass accuracy lower than 1 ppm. The instruments were controlled using the Xcalibur software (Thermo Fisher Scientific). Metabolites were sought based on the calculated m/z of the corresponding pseudo-molecular ion [M + H]^+^ in positive mode and [M–H]^−^ in negative mode from a list of metabolites of interest using a suspect screening approach (Krauss et al., 2010; Flamini et al., 2013).

Putative metabolite identifications were proposed based on expertized analysis of the corresponding mass spectra and comparison with published literature. Further information was retrieved from the Kyoto Encyclopedia of Genes and Genomes (KEGG1) and PubChem2 databases. The identification of some metabolites was confirmed with the corresponding standard for: amino-acids (arginine, asparagine, glutamine, glutamate, aspartate, valine, proline, leucine, tyrosine, isoleucine, phenylalanine, tryptophane, and valine), phenolic acids (gallic acid and caffeic acid), flavonoids (catechin, epigallocatechin gallate, and naringenin) and stilbenoids (*trans-*resveratrol, pterostilbene, and ɛ-viniferin). All standards were provided by Sigma-Aldrich (France). Stilbene oligomers for which no standard was available were putatively identified as stilbene dimer M471, stilbene dimer M473, stilbene trimer M679, stilbene trimer M681, stilbene tetramer M907, where MXXX stands for the *m/z* of the corresponding ion in positive mode. Relative quantification of the selected metabolites was performed using the Xcalibur software.

Differential metabolomic analyses were performed after log10 data transformation, using Tukey’s honest significant difference method followed by false discovery rate (FDR) correction using the Benjamini-Hochberg procedure. Metabolites of interest were considered differentially accumulated when the false discovery rate was below 5% (FDR < 0.05).

### Proteomic samples preparation

At 1 and 3 months of culture, for beech and grapevine cultures, extracellular proteins were extracted from three flasks per culture condition. Each flask was agitated with 30 ml of 30 mM potassium-acetate buffer at pH 4.5 for 2 h at 60 rpm at room temperature. Extracts were centrifuged 20 min at 4,000 rpm at 4°C and supernatants were precipitated overnight at −20°C in cold 80% acetone. Precipitated proteins were dried after centrifugation 20 min at 4,000 rpm at 4°C and diluted in 40 μl of concentrated Laemmli blue (2-mercaptoethanol 24% (*w*/*v*), bromophenol blue 17.3 M, glycerol 20% (*v*/*v*), sodium dodecyl sulfate 10% (w/v) and Tris-base 0,4 M). Samples were heated for10 minutes at 95°C and 15 μl of each sample were deposited on 1 mm 15% SDS-PAGE gels for short-migration. Gels were revealed with Coomassie blue coloration and gel pieces were sent for proteomic analyses.

### Proteomic analysis

Proteomic analyses were achieved by PAPPSO platform, INRAE, Jouy-en-Josas. The analyses of peptides were obtained using an UltiMate^™^ 3,000 RSLCnano System (Thermo Fisher Scientific, San Jose, CA, United States) coupled to an Orbitrap Fusion^™^ Lumos^™^ Tribrid^™^ mass spectrometer (Thermo Fisher Scientific). Samples were digested with trypsin and resuspended in 2% ACN and 0.1% TFA buffer. Four μl were injected on an Acclaim PepMap C18 precolumn (5 μm × 300 μm × 5 mm) at 20 μl/min and separated on an Acclaim PepMap RSLC nanoViper C18 column (2 μm × 75 μm × 500 mm, Thermo Fisher Scientific). Mobile phase was composed of A: 98% H_2_O in 0.1% formic acid (HCOOH) and B: 80% ACN in 0.1% HCOOH. A gradient from 1.0% B during 2 min, to 30% B for 48 min and to 40% B for 5 min, followed by regeneration step at 98% B in 2 min for 5 min and equilibration at 1.0% B in 0.5 min for 4.5 min, was applied at 300 nl/min. Peptide ions were analysed using Xcalibur 4.1, Tune 3.0. with the following parameters: MS scan m/z 400–1,500 and resolution 120,000; MS/MS collision energy 30%, resolution 30,000, and dynamic exclusion set at 100 s. RAW data were converted to mzXML data with proteowizard software (Chambers et al., 2012). Proteins annotations was performed using the X!TandemPipeline (Langella et al., 2017) and https://mycocosm.jgi.doe.gov/Fomme1/Fomme1.home.html, https://mycocosm.jgi.doe.gov/Trave1/Trave1.home.html and an internal contaminants database. Only proteins with at least two distinct peptides were considered. Proteins relative abundancies were compared using protein abundance index (PAI) and exponentially modified protein abundance index (emPAI; Ishihama et al., 2005). Proteins with emPAI higher than 10 were selected for comparative analysis and were functionally annotated according to Joint Genome Institute (JGI) online database.

### Statistical analyses

Data analysis and graphs were done using Rstudio, PBC software version 2022.02.3. Shapiro–Wilk test was used for data normality check when relevant. Kruskal-Wallis tests with Conover-Iman multiple comparisons test were used for nonparametric tests and Student Newman–Keuls (SNK-test) for parametric tests. All significant differences are indicated by ^***^ for *p*-values <0.001, ^**^ for *p*-values < 0.005 and ^*^ for *p*-values < 0.05.

## Results

**Figure 2:**
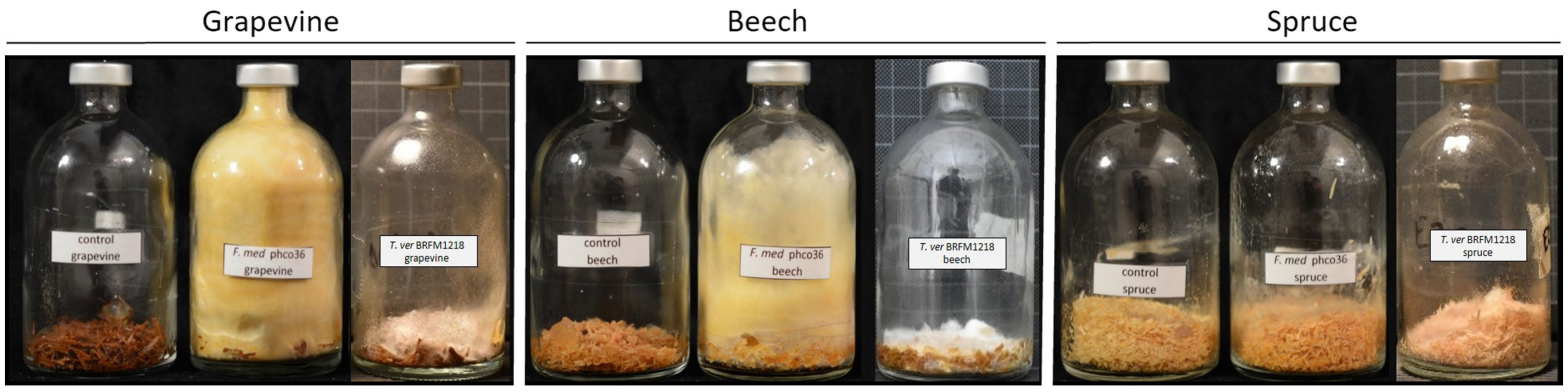
Control, Fmed (*F. mediterranea* phco36) and Tver (*T. versicolor* BRFM1218) cultures on grapevine, beech and spruce at the end of the cultures. Grapevine and beech cultures were stopped at 3 months, and spruce cultures were stopped at 2 months. A yellow and wolly mycelium was observed for Fmed whereas Tver mycelium remains white and at the bottom of the flask. On spruce, a slight coloration of sawdust and white mycelium are observed with Fmed, and no mycelum is observed with Tver.

Fmed and Tver were grown on sawdust from three different wood: grapevine cv. ‘Gewurztraminer’, beech, and spruce, during 3 months at 27°C in 120 ml flasks (Figure 2). Flasks were hermetically closed, and oxygen was renewed every 3–4 days. At each oxygen renewal, the amount of produced and accumulated CO_2_ was measured, to follow fungal growth and wood degradation in a non-destructive way. Spruce sawdust cultures were stopped after 2 months because no more carbon mineralization was measured before the end of the 2-months spruce cultures. As shown in Figure 1, for each test condition, at least 12 replicates or flasks were inoculated at the same time. Among them, three replicates were sampled at time 0, 1 month, 2 months (for spruce and grapevine) and 3 months (for grapevine and beech) for mass loss, methanolic extraction (for metabolomic analyses) and wood fibers analyses. Three other replicates were sampled at 1 and 3 months of culture for proteomic analyses, for grapevine and beech only as only low carbon mineralization and no mass loss were observed on spruce.

### Morphological observations

Both Fmed and Tver showed distinct morphologies, as shown in Figure 2. On grapevine and beech sawdust, Fmed had the same morphology: white mycelium at the beginning, turning yellow while colonizing the whole volume of the flask. On the contrary, the Tver mycelium remains white and at the bottom of the flask, on the sawdust. On spruce sawdust, a light and white mycelium was observed with Fmed, as well as a slight change in sawdust color (darkening). No mycelium was observed in the spruce Tver culture. A light white fruiting bodies were observed in any of the cultures.

### Carbon mineralization rate

**Figure 3:**
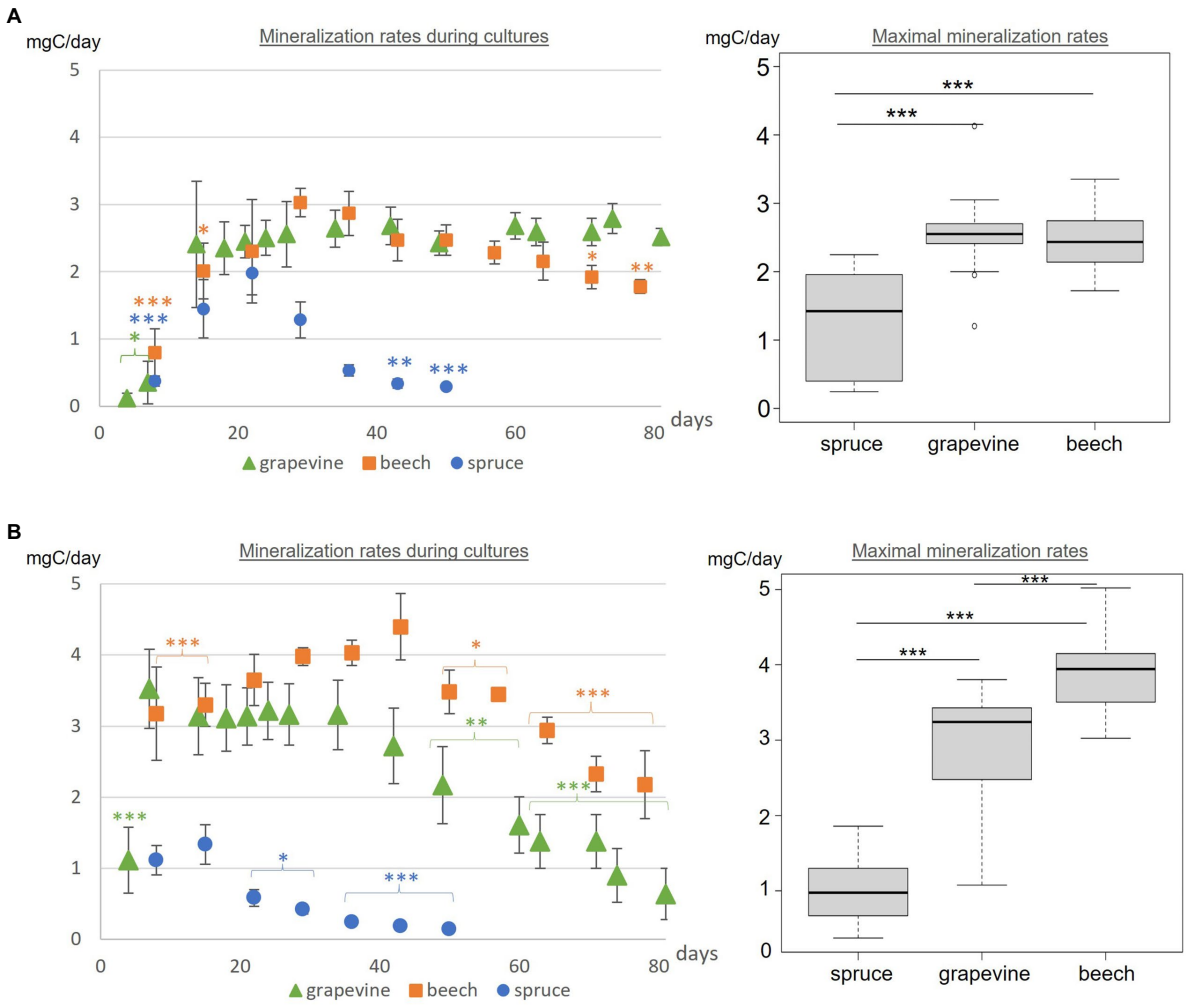
**(A)** Fmed and **(B)** Tver carbon mineralization rates during cultures and maximal respiration rates for grapevine, beech and spruce cultures. Statistics between mineralization rates during cultures are indicated for each point compared to the highest measured value in the same culture with Tukey test. Statistical differences between maximal mineralization rates were obtained with Tukey test. Significant differences are indicated by *** for *p*-values < 0.001, ** for *p*-values < 0.005 and * for *p*-values < 0.05.

Carbon mineralization rates were obtained by measuring the CO_2_ content (%*v*/*v*) and expressed in mgC/day (Figure 3A). CO_2_ content was renewed to atmospheric content after each measurement. Carbon mineralization rates of Fmed cultures differed depending on the substrate. On grapevine, a rapid increase was observed (before day 15th), followed by a stabilization until the end of the culture (84 days). On beech sawdust, three phases of the mineralization rate were observed: an increase (before day 22nd), a stabilization and a decrease (after day 63rd). Increase phase was longer (or slower) compared to that observed on grapevine. On spruce sawdust, the Fmed mineralization rate increased before day 22nd and decreased after day 36th.

The maximal mineralization rate corresponds to the mean mineralization rate during the stabilization phase. For the latter, no differences were observed between Fmed grapevine or beech cultures (2.55 and 2.46 mgC/day respectively). A lower maximal mineralization rate was observed for Fmed spruce culture (1.27 mgC/day).

In Tver cultures, with the three substrates, three phases of the mineralization rate were successively observed: an increase, a stabilization and a decrease (Figure 3B). On grapevine and spruce, the increase phase occurred before day 7th whereas it occurred before day 22nd on beech. On grapevine and beech, the decrease phase started after 43 incubation days while it started after 15 incubation days on spruce. Contrary to Fmed cultures, a higher maximal respiration rate was observed in Tver beech culture than in Tver grapevine culture. For both substrates, this rate was significantly higher compared to Fmed cultures (*p*-values < 0.001). Tver and Fmed maximal mineralization rates were similar on spruce.

### Mass loss

**Figure 4:**
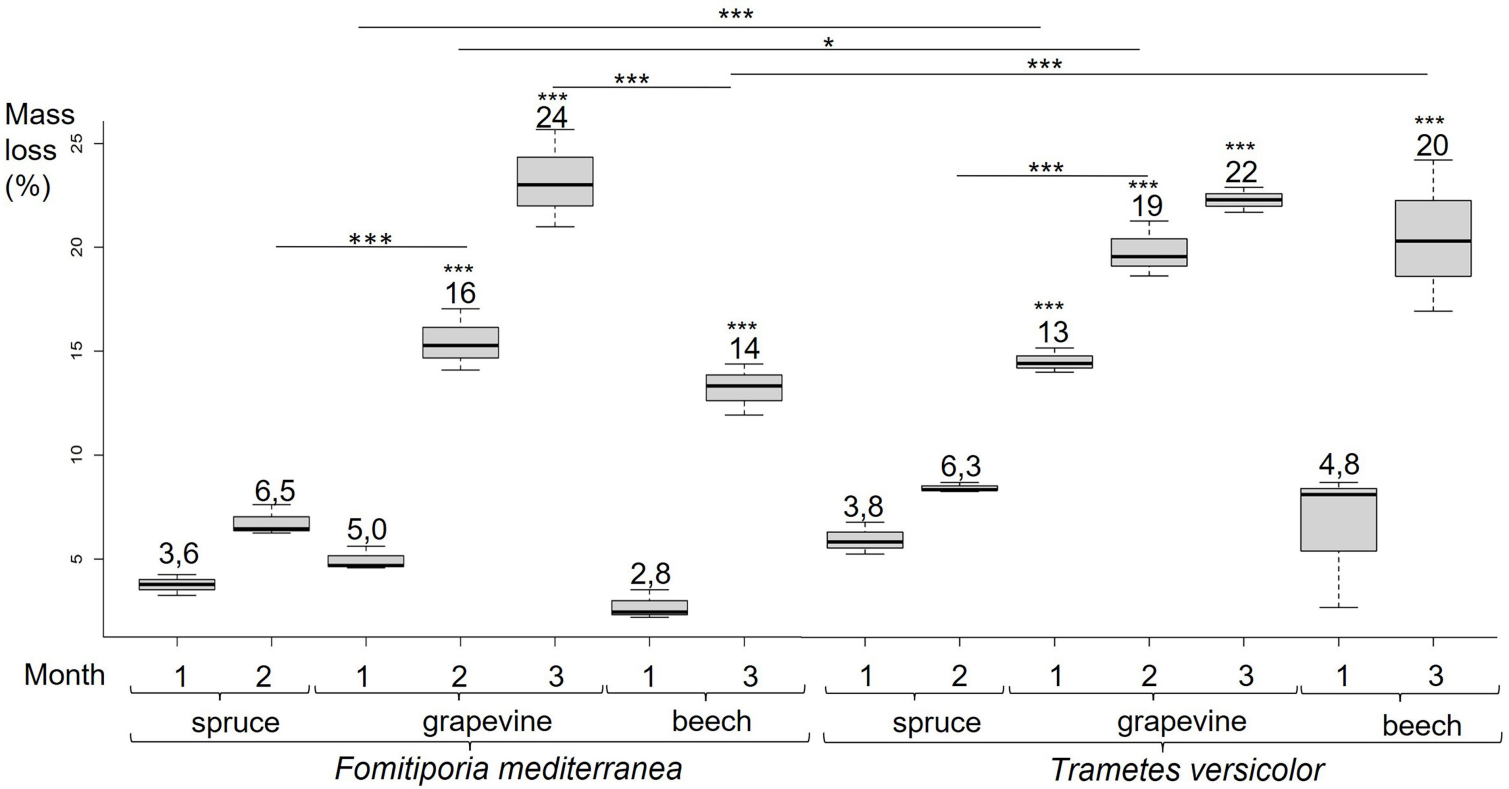
Fmed and Tver cultures mass loss on spruce at 1 and 2  months, grapevine at 1, 2 and 3  months, and beech at 1 and 3  months. Significant differences obtained with Conover-Iman test of multiple comparisons are indicated by *** for *p*-values < 0.001, ** for *p*-values < 0.005 and * for *p*-values < 0.05.

Global mass loss was obtained from cultures total dry weight at 0, 1, 2 or 3 months of culture (Figure 4). After 1 month of fungal exposure to Fmed, no significant mass loss was observed for any of the three sawdust. After 2 months, Fmed culture on spruce caused no significant mass loss, whereas 15.7% of mass loss was observed on grapevine. After 3 months, a higher mass loss (23.6%) on grapevine than on beech (13.8%) was observed. Thus, consistently with mineralization rates, Fmed did not significantly degrade spruce wood and degraded more grapevine wood than beech wood.

After 1 and 2 months of incubation in the presence of Tver, mass losses measured in grapevine wood cultures (13.3 and 19.0% respectively) were higher than with Fmed. As for Fmed cultures, no significant mass losses were observed on spruce after 2 months of exposure to Tver. Unlike the two first months, a similar degradation was observed with both fungi after 3 months of grapevine culture (21.7% with Tver). A similar mass loss was observed on beech cultures after 3 months of exposure to Tver (20.3%). Thus, after 3 months, Fmed and Tver degraded an equivalent amount of grapevine wood and Fmed degraded less beech wood than Tver.

### Wood fibers analyses

**Figure 5:**
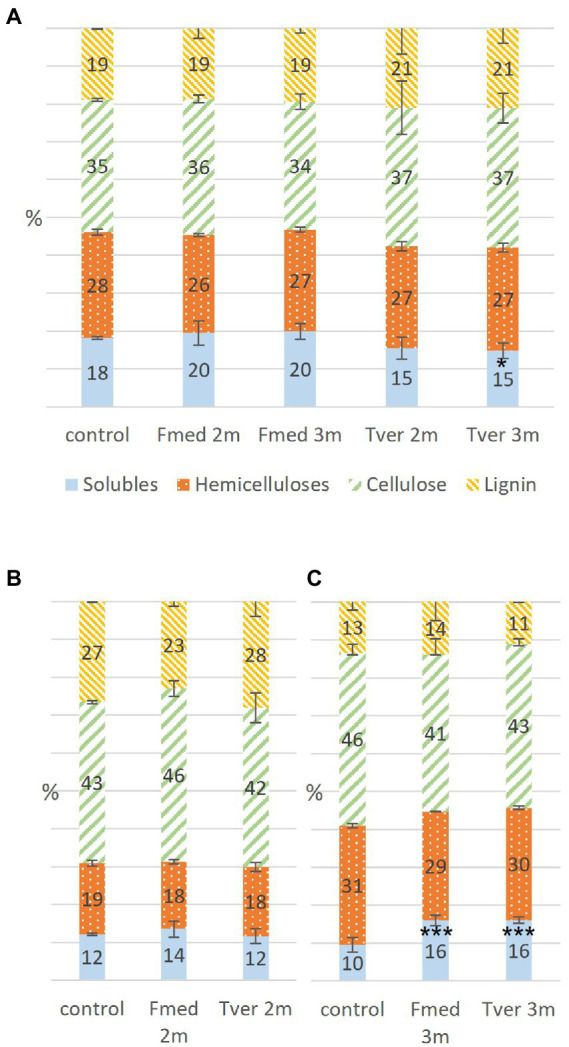
Proportions in wood components in control, Fmed and Tver cultures on **(A)** grapevine, **(B)** spruce and **(C)** beech at 1  month (1 m), 2  months (2 m) or 3  months (3 m) of culture. Significant differences are given compared to the percentage in control according to Student–Newman–Keuls test, and are indicated by *** for *p*-values < 0.001, ** for *p*-values < 0.005 and * for *p*-values < 0.05.

Soluble compounds, hemicelluloses, cellulose, and lignin contents were obtained at 0, 1, 2 and/or 3 months of each culture according to Van Soest method (Van Soest and McQueen, 1973). Composition of control wood sawdust was the same at each month of culture, including time 0. Mean composition of control sawdust and sawdust exposed to fungal culture during 2 or 3 months are shown in Figure 5A for grapevine, Figure 5B for spruce and Figure 5C for beech. Grapevine initially contained more lignin (19%) than beech (13%) and less than spruce (25%); less hemicelluloses (28%) than beech (31%) and more than spruce (19%); less cellulose (35%) than both beech (46%) and spruce (43%); and the highest soluble compounds content (18%) compared to beech (10%) and spruce (13%). After 2 and 3 months of fungal exposure to both Fmed and Tver, no significant variations regarding cellulose, hemicelluloses and lignin were observed for all substrates. Concomitantly, after 3 months of fungal culture, soluble compounds content increased in beech sawdust exposed to Fmed and to Tver (+6%). In grapevine sawdust exposed to Fmed during 2- and 3-months, this content remained equivalent, whereas it decreased after 3 months exposed to Tver (−3%).

### Targeted metabolomic analysis

**Figure 6:**
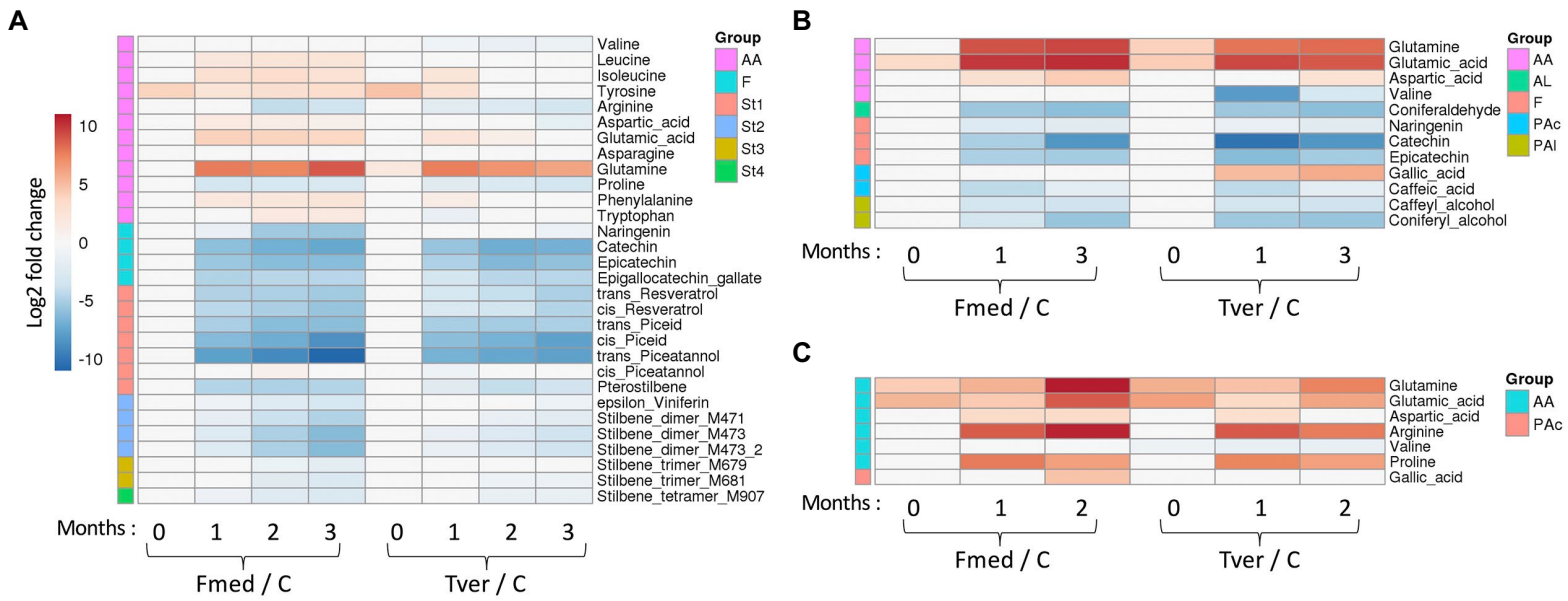
Targeted metabolomic analysis of the wood degradation process. Log2 of significant metabolite fold changes for indicated pairwise comparisons are given by shades of red or blue colors according to the scale bar for **(A)** grapevine, **(B)** beech and **(C)** spruce cultures. Data represent the mean values of three biological replicates for each condition and time point. Statistical analyses were performed by Tukey’s honest significant difference method followed by false discovery rate (FDR) correction, with FDR < 0.05. For FDR ≥ 0.05, Log2 fold changes were set to 0. AA: amino acids; AL: aldehydes; F: flavonoid; PAc: phenolic acids; PAl: phenolic alcohols; St1 to St4: Stilbene monomers, dimers, trimers and tetramers; MXXX: *m/z* in positive mode. C: control.

Fourteen stilbenes and four flavonoids were detected with grapevine cultures (Figure 6A), three flavonoids with beech cultures, and no wood extractives on spruce cultures (Figure 6C), whereas in total twelve amino acids (AA) and five other phenolic compounds were differentially detected (Figure 6). Stilbene oligomers, for which no standards were available, were putatively identified as stilbene dimers, trimers and tetramers as indicated. Heatmaps of relative mean peak areas of detected metabolites for each wood are given in Supplementary Figure S1. The latter shows that no time effect was observed, as no quantitative variations were observed between 0-, 1-, 2-, and 3-months control cultures.

Significant variations in the relative amounts of the selected metabolites were observed between the control and the culture in a time-dependant manner, as shown in Figure 6. During Fmed grapevine and beech cultures, the content of all selected flavonoids and stilbenes decreased. Epigallocatechin gallate, catechin, *trans-* and *cis-*resveratrol, *trans-* and *cis-*piceid, *trans-*piceatannol, pterostilbene and two stilbene dimers (M471 and M473) contents decreased from the first month of culture. Relative amount of naringenin, *cis-*piceatannol, ε-viniferin, one stilbene trimer and one stilbene tetramer (M681 and M907) decreased from 2 months of culture, and one stilbene trimer (M679) content decreased after 3 months of culture. Epicatechin content decreased at 1 month and reverted to control value at 2 and 3 months. A very slight natural degradation of this compound in control samples (Supplementary Figure S1), or an increase of epicatechin content during the second month in presence of Fmed. In beech cultures, catechin, epicatechin and naringenin contents also decreased after the first month of culture. Thus, most of the selected extractives were degraded by Fmed after different culture times.

Not all extractive contents in Tver grapevine culture decreased to the same extent, some of them decreasing less intensely or slower than in Fmed culture. Three stilbenes, *cis*-piceatannol and the two stilbene trimers (M679 and M681), were not degraded. All other grapevine extractives, except catechin, epicatechin, *cis*-piceid and *trans*-piceatannol were less or more slowly degraded compared to Fmed cultures.

Besides extractives, primary metabolites contents in Fmed cultures evolved differentially depending on the wood species. With grapevine, an increase in eight AA levels was observed (isoleucine, aspartate, glutamate and glutamine after 1 month and leucine, tyrosine, phenylalanine and tryptophane after 2 months). In beech and in spruce cultures, only three of them (glutamine, glutamic acid, and aspartate) increased. Proline and arginine levels decreased in grapevine cultures after 1 and 3 months, respectively. Interestingly, in spruce cultures an increase of arginine and proline content was observed.

When growing Tver on grapevine sawdust, differences between fungal cultures and control have been observed for only three AA: glutamine level increased after 1 month, proline level decreased after 2 months, and valine level decreased after 1 month. Valine level decreased in beech cultures exposed to Tver for 1 month, and not when exposed to Fmed. AA levels variations were similar in beech and spruce cultures exposed to both Fmed and Tver.

### Proteomic analysis

**Figure 7:**
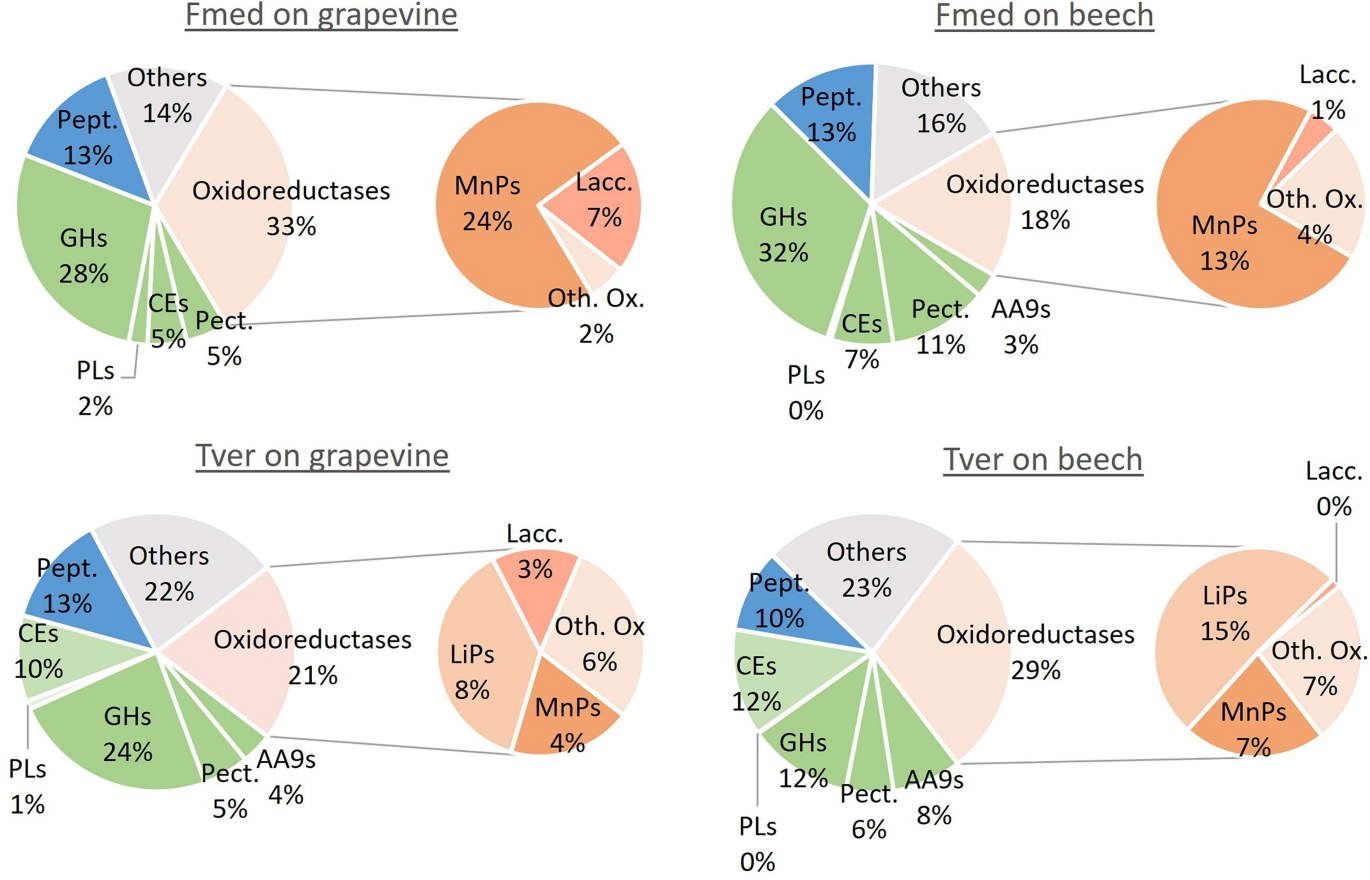
Relative abundance of main classes of proteins in Fmed and Tver secretome on grapevine and beech cultures. Pept.: peptidases, GHs: glycoside hydrolases, PLs: polysaccharides lyases, CEs: carbohydrate esterases, Pect.: pectinases, AA9 = auxiliary enzymes family 9 (or LPMOs), MnPs: manganese peroxidases, Lacc.: laccases LiPs: lignin peroxidases, Oth. Ox.: other oxidoreductases. Classification was performed according to JGI database and percentages are given according to PAI relative abundancies for all proteins with emPAI higher than ten in at least one of the culture conditions.

Proteomic analyses were conducted on 1- and 3-months grapevine and beech cultures of Fmed and Tver. Proteins with emPAI above 10 in at least one experimental condition were selected for comparative analysis. Thus, respectively 113 and 122 proteins were compared in Fmed and Tver secretomes (Table 1). As expected, mainly extracellular proteins were detected, except for two P450s in Fmed cultures (Supplementary Table S1). As shown in Table 1, a similar amount of secreted proteins were found in Fmed grapevine (101 proteins) and beech cultures (93 proteins). MnPs were the only class-II peroxidases detected. Among the 16 MnPs identified in Fmed genome (Floudas et al., 2012), 11 were found on grapevine wood whereas eight were found on beech. Three and two laccases were found on the two substrates, respectively, among the ten identified in the Fmed genome (Floudas et al., 2012). Few differences regarding the number of CAZYs detected in the Fmed secretome on grapevine or beech were observed (50 on grapevine and 49 on beech). Also, more peptidases were found with grapevine wood (18) compared to beech wood (11). Relative abundances for secreted proteins in Fmed and Tver grapevine and beech cultures are shown in Figure 7. More oxidoreductases were found with grapevine (33%) rather than with beech (18%). Among them, mainly MnPs were found (respectively 24 and 13% of total proteins content). More laccases were found with grapevine (7%) than with beech (1%). Protease abundancy was also higher with grapevine (14%) than with beech (10%). Thus, more MnPs, laccases and peptidases were observed in the Fmed secretome on grapevine compared to beech sawdust, both regarding protein diversity and abundancy. Total numbers and abundancies of CAZYs were similar between the two substrates, and few differences in CAZYs composition were observed (Supplementary Table S1).

**Table 1:** Number of detected proteins functionally classified in Fmed and Tver secretomes on grapevine and beech wood sawdust in 1- and 3-months cultures.

Protein families	Fmed								Tver							
	Genome^1^	All	Grapevine wood			Beech wood			Genome^1^	All	Grapevine wood			Beech wood		
			All	1 month	3 months	All	1 month	3 months			All	1 month	3 months	All	1 month	3 months
Oxidoreductases																
MnP	16	11	11	10	7	8	8	4	13	6	5	5	0	3	2	2
LiP	0	0	0	0	0	0	0	0	10	8	8	8	1	7	7	0
VP	0	0	0	0	0	0	0	0	3	0	0	0	0	0	0	0
GP	1	0	0	0	0	0	0	0	0	0	0	0	0	0	0	0
Total Class II-POD	17	11	11	10	7	8	8	4	26	14	13	13	1	10	9	2
DyP	3	0	0	0	0	0	0	0	2	1	1	1	1	1	1	1
HTP	4	0	0	0	0	0	0	0	3	0	0	0	0	0	0	0
Laccases “*sensu stricto*”	10	3	3	3	2	2	2	0	7	4	3	3	1	1	0	1
Ferroxidases	1	0	0	0	0	0	0	0	2	0	0	0	0	0	0	0
Ferroxidase/laccase	0	0	0	0	0	0	0	0	1	0	0	0	0	0	0	0
Glyoxal oxidase	0	1	1	0	1	1	0	1	5	4	2	1	2	4	4	4
Copper radical oxidase	4	0	0	0	0	0	0	0	4	0	0	0	0	0	0	0
CDH	1	0	0	0	0	0	0	0	1	1	1	1	0	0	0	0
Other GMC oxidase	4	4	1	1	1	4	4	3	9	0	0	0	0	0	0	0
Total oxidoreductase	44	19	16	14	11	15	14	8	60	24	20	19	5	16	14	8
CAZYs																
GH	50	43	40	21	38	38	27	33	47	45	42	35	36	34	30	30
AA9	13	2	2	2	2	2	2	2	18	5	4	4	1	3	3	3
CE	12	8	7	3	6	8	7	8	14	8	6	5	6	8	5	8
PL	?	1	1	1	1	1	1	0	?	1	1	0	1	0	0	0
Total CAZY	75	54	50	28	47	49	37	43	79	59	53	44	44	45	38	41
incl. CBM	?	8	8	6	7	7	5	5	?	13	11	11	10	11	9	10
Others																
Peptidases/proteases	–	18	15	10	14	11	7	11	–	14	12	10	9	8	8	6
Others or unknown	–	22	20	7	18	18	13	16	–	25	20	14	14	16	8	16
TOTAL	–	113	101	58	90	93	71	78	–	122	105	87	72	85	68	71

1 Genomic data were found in Floudas et al. (2012).

Proteins for which emPAI relative abundancy was higher than ten in at least one of the culture conditions were selected. MnP: manganese peroxidase, LiP: lignin peroxidase, VP: versatile peroxidase, GP: generic peroxidase, Class-II-POD: class-II peroxidase, DyP: dye-decolorizing peroxidase, HTP: heme-thiolate peroxidase, CDH: cellobiose dehydrogenase, GMC: glucose-methanol-choline oxidoreductase, GH: glycoside hydrolase, AA9: auxiliary enzyme family 9 (syn. LPMO: lytic polysaccharide monooxygenase), CE: carbohydrate esterase, PL: polysaccharides lyase, CAZY: carbohydrate active enzyme, incl. CBM: including cellulose binding module.

In the Tver genome, eight LiPs and six MnPs were identified, among, respectively, 10 and 13 identified in the Tver genome (Floudas et al., 2012), whereas no VPs nor GPs were detected either on grapevine or on beech (Table 1). On grapevine and on beech, eight and seven LiPs and five and three MnPs, respectively, were identified In the Tver secretome. Four laccases on the seven identified in the Tver genome (Floudas et al., 2012) were found on grapevine and one on beech. However, as shown in Figure 7, oxidoreductases were less abundant on grapevine than on beech. Moreover, contrary to the Fmed secretome (Supplementary Figure S2A), CAZYs abundancies in Tver secretome showed substrate specificities for some of these proteins (Supplementary Figure S2B). Indeed, for the latter, proteins from GH15 and GH43 families were detected with higher relative abundancies on grapevine compared to beech cultures. One alpha-amylase from GH13 family subfamily 32, one GH17 and one glucosidase from GH31 family were more represented on grapevine than on beech cultures. On the contrary in the Fmed secretome, GHs from those families were either not found (no GH13 identified) or found on both grapevine and beech. Some GH families were identified only in Fmed or in Tver secretome (Supplementary Table S2), such as GH1 or GH18 only detected in Fmed secretome.

## Discussion

Important mechanisms involved in Fmed wood degradation and detoxification were described. A simultaneous degradation pattern, i.e., the concomitant degradation of all wood structure components, was observed with the two hardwood species, while no significant mass loss was observed with spruce. In parallel, specificities of Fmed mechanisms on grapevine wood were highlighted, namely regarding carbon mineralization rate, mass loss, metabolomic analyses and secreted proteins analyses.

Wood initial compositions of the three substrates were different: grapevine showed low cellulose contents and high soluble compounds contents. Its lignin content was lower compared to spruce and higher compared to beech. A decrease of soluble compounds was observed in grapevine cultures while their content increased in beech cultures. Soluble compounds include extractives and other non-structural wood compounds, such as soluble nutriments, sugars or nitrogen reserves. Degradation or consumption of the latter could lead to soluble compounds decrease, whereas accumulation of fungal wood degradation products or soluble compounds from mycelium could lead to an increase. Further experiments such as more precise determination of the nature of soluble compounds would give more information about fungal growth mechanisms. In parallel, the absence of variations in proportions of the three main structural components suggests a simultaneous WRF degradation pattern (Blanchette et al., 1985). This was observed on both grapevine and beech wood. This is consistent with one previous study based on wood fibers analysis (Pacetti et al., 2022). Initial lignin and soluble contents in grapevine wood (respectively, 19 and 18%) are higher than previously reported for the same *cv.* (15.7 and 14.6%), while measured hemicelluloses content is lower than previously reported (respectively, 28 and 35.7%; Pacetti et al., 2022). In another study with grapevine cv. Sangiovese, higher lignin and lower hemicellulose contents were observed in brown streaking wood (17.2%) and brown red wood (29.9%) compared to asymptomatic wood (12.1%; Agrelli et al., 2009). Previous fungal degradation, for example by the two Ascomycota *P. chlamydospora* and *P. minimum* associated with brown necrosis, could explain a higher lignin content (Agrelli et al., 2009). Several other parameters could affect lignin content in wood, such as environmental stresses (Blanchette, 1991). For this study, only white parts of wood, considered as healthy, were selected. Fmed ability to degrade healthy grapevine wood is consistent with previous studies showing that the latter does not require previous degradation, namely by the two Esca-associated Ascomycota *P. chlamydospora* and *P. minimum* (Sparapano et al., 2001; Brown et al., 2020). However, among other factors, different structural compositions were shown to induce different fungal wood degradation patterns (Blanchette, 1991). For example the WRF *Pleurotus ostreatus* shows simultaneous degradation with beech and lignin preferential degradation with oak wood (Bari et al., 2020). Our results show simultaneous degradation pattern on beech and grapevine wood for both Fmed and Tver. This is consistent with *in vitro* and *in vivo* studies showing a simultaneous degradation for Tver on beech, spruce, oak or hornbeam (Karim et al., 2017; Bari et al., 2020). Fmed wood degradation patterns might be different with other substrates, such as other wood species or previously degraded wood, or in different growth conditions. Fmed was identified on different wood species, such as *Actinidia* sp., *Citrus* sp. or olive trees (Moretti et al., 2021) and strains specificities were shown for Fmed depending on the wood species from which they were isolated (Markakis et al., 2017). Thus, wood degradation pattern might be more specific upon other wood species and investigation with more Fmed strains and more wood species could highlight more Fmed specificities.

Contrary to wood degradation pattern, other parameters suggest a specific activity for Fmed on grapevine cultures compared to the other substrates: a longer stabilization phase for maximal mineralization rate, a higher mineralization rate (compared to spruce only), higher mass loss, more secreted laccases and MnPs and elevated AA levels. Indeed, carbon mineralization rate on grapevine cultures reached a plateau and maintained it until the end of the 3-month cultures. On the contrary with beech and spruce cultures, mineralization rates decreased before the end of the cultures. CO_2_ production or carbon mineralization results from metabolic processes including fungal respiration. With wood as the only carbon source, carbon mineralization rate can be considered as an indicator of wood degradation. The first increase of carbon mineralization rate followed by a plateau was already observed in fungal cultures with 18 white or brown rot species isolated from forest softwoods with different microclimatic preferences (Carlsson et al., 2017). The authors suggested that fungi first grow exponentially while colonizing the whole substrate, and then reach a substrate-species equilibrium. Thus, a longer equilibrium phase suggests a better fungal growth on this substrate. Moreover, the speed of colonization phase and the maximal mineralization rate are specific to fungal species, and can be related to different fungal colonization strategies (Boddy and Heilmann-Clausen, 2008; Carlsson et al., 2017). Here should be noticed that respiration might not only result from wood degradation but also from fungal storage compounds metabolization or self-recycling processes (Hiscox et al., 2015). Moreover, MnPs activities were also shown to produce CO_2_ (Hofrichter et al., 1999). Thus, carbon mineralization rates are not only related to fungal growth but also to fungal metabolic activity. The latter is higher for Fmed with the two hardwoods species compared to spruce wood, and a longer equilibrium phase is observed with grapevine wood compared to beech.

As suggested by carbon mineralization rate, higher mass loss on grapevine cultures compared to beech and spruce confirm a higher wood degradation rate for Fmed on grapevine. This degradation rate is higher than the one reported by a previous study (10% after 3-months) using the same fungal strain and grapevine *cv.* (Pacetti et al., 2022). In the latter study grapevine wood blocks (0.5 × 5 × 2.5 cm) were used and, among other parameters, the three-dimensional structure of wood initial wood composition might have limited fungal access to wood polymers (Blanchette, 1991). On spruce wood, mass losses are lower than on the two hardwoods for both fungi. Thus, spruce wood appears as more recalcitrant to fungal degradation, which is consistent with previous studies (Bari et al., 2020). This can be explained by the higher lignin content observed in spruce, or by softwood lignin composition mainly containing guaiacyl units (Blanchette et al., 1988). Wood degradation rates between beech and grapevine for Tver cultures were similar, contrary to Fmed cultures suggesting that this latter is less efficient to degrade beech wood than grapevine wood. This could be at least partially explained by the nature of Class-II PODs secreted by the two fungi. Indeed, consistently with genomic data (Floudas et al., 2012), MnPs were the only class-II PODs detected in the Fmed secretome, whereas both LiPs and MnPs were detected in the Tver secretome. Moreover, more MnPs and laccases were secreted by Fmed on grapevine than on beech. Enhancement of MnPs and laccases gene expression in mycelium exposed to wood compared to control cultures were previously described (Pacetti et al., 2022). The highest wood degradation rate with Tver on beech cultures compared to Fmed could be related to LiPs capacity to degrade non-aromatic parts of lignin, additionally to aromatic parts, contrary to MnPs (Wong, 2009). Our results show a positive correlation between wood degradation rate by Fmed and MnPs relative abundancies. This observation is consistent with the role of those enzymes in wood degradation. However, this is not the case in Tver cultures. A similar degradation rate with both substrates by Tver is associated with more oxidoreductases on beech than on grapevine, suggesting a more important role of CAZYs in grapevine wood degradation by Tver. Higher abundancies of some GHs families, such as GH15 or GH43, could be involved in specific degradation. Indeed, specific carbohydrate degradation activities upon the woody substrate were shown for the white-rot species *Dichomitus squalens*: a higher xylanolytic activity on hardwood (birch) than on softwood (spruce), which are containing, respectively, more xylan and more mannan (Daly et al., 2018). Xylose was reported as the main sugar found in hemicelluloses of grapevine pruning wood (Nitsos et al., 2016), suggesting a majority of xylan and/or xyloglucan. This is also the case for beech wood, consistently with common hardwood hemicellulose composition (Laine, 2014; Ibrahim and Kruse, 2020). On the contrary, no clear differences were observed between the two substrates for CAZYs composition in Fmed secretome upon the substrate. Thus, Fmed modulates its oxidoreductases secretion upon its substrate but not the nature of GHs. Changes in secretome linked to environmental factors have been well described, although regulation mechanisms remain not fully understood (McCotter et al., 2016). Several factors such as hemicellulose or lignin composition, pH or wood extractives might have induced changes in the two WRF secretomes. Tver secretome modulations were associated to similar wood degradation rates, whereas Fmed degraded grapevine wood more efficiently than beech wood. Thus, Fmed might be more specifically adapted to grapevine wood compared to other wood species.

Targeted metabolomic analysis aimed at following wood extractives during exposure to Fmed or Tver and eventually highlighting fungal metabolic activity through primary metabolite detection. All selected extractives, stilbenes and flavonoids in grapevine wood and flavonoids in beech wood, decreased during Fmed cultures. Stilbenes are known for their biological activities and for their accumulation in grapevine wood in response to a pathogen attack (Galarneau et al., 2021). Their presence and levels in wood depend on several factors, including grapevine cultivar (Gabaston et al., 2019). Gewurztraminer and Pinot noir were reported as particularly rich in stilbenes compared to other cultivars, with total stilbenes content ranging from 4,628 to 5,830 mg/kg DW (Vergara et al., 2012; Lambert et al., 2013; Guerrero, 2016). In our wood material ɛ-viniferin, the most abundant, *trans*-resveratrol and piceatannol were identified, which is in accordance with the reported stilbene composition of *cv.* Gewurztraminer (Vergara et al., 2012; Lambert et al., 2013; Guerrero, 2016). Six grapevine stilbenes (resveratrol, pterostilbene, miyabenol C, vitisin A and B and isohopeaphenol) were shown to partially inhibit Fmed growth (Bruno and Sparapano, 2006b; Lambert et al., 2012). Fmed resveratrol detoxification activity was demonstrated *in vitro* (Bruno and Sparapano, 2006b), and the decrease of resveratrol content in Fmed cultures is consistent with this observation. More efficient detoxification processes might enhance wood degradation. Indeed, grapevine extractives were degraded faster and to a higher extent in presence of Fmed than of Tver. Interestingly, more laccases were detected in Fmed secretome in 1-month cultures compared to 3-months cultures. In addition, almost all extractives were already degraded in 1-month cultures. This is consistent with known potential detoxification activities of laccases (Jönsson et al., 1998; Lekounougou et al., 2008; Valette et al., 2017). Our results suggest a fungal-substrate adaptation, as several extractive contents in grapevine wood (*cis*-piceatannol, stilbene dimers, trimers and tetramer, including ɛ-viniferin, and naringenin) decreased faster and to lower levels in Fmed cultures compared to Tver cultures, and the opposite was observed for catechin in beech cultures.

Variations of amino acids levels have been observed depending on the used fungus and substrate. AA are found as nitrogen reserves in grapevine, and arginine was reported as the most represented (Zapata et al., 2004). In grapevine cultures exposed to Fmed or to Tver, arginine and proline levels decreases might be due to fungal degradation or metabolization. In previous studies AA levels variations during grapevine-fungi interactions were attributed to grapevine reaction, as experiments were conducted on living wood (Labois et al., 2021). In our conditions, they can be rather attributed to fungal metabolic activity. AA are essential for various fungal metabolic pathways, and their levels increase is consistent with the presence of proteases observed in both Fmed and Tver secretomes. They might be due to degradation products from wood proteins or to fungal autolysis processes. Differences observed in the nature and the levels variations of AA, depending on the cultures, suggest fungal-substrate specific activities.

To conclude, Fmed was able to degrade two hardwood species namely grapevine, a liana on which it is involved in wood disease, and beech, a forest tree. Simultaneous degradation patterns were observed on both substrates, like a majority of WRF including Tver, the comparison model used in this study. Fmed specific traits on grapevine were highlighted: a higher degradation rate than with beech, more laccases and MnPs secreted, and a more efficient extractive detoxification compared to beech and compared to Tver. Thus, Fmed wood degradation mechanisms seem to be more adapted to grapevine wood than to other woods such as beech or spruce. However, the ubiquitous WRF Tver was able to modulate its secretome and to degrade grapevine wood as efficiently as Fmed, although it has never been observed on this species. Thus, beside its ability to degrade grapevine wood, the widespread of Fmed in vineyards may rely on other factors. For example, adaptation to higher extractive contents in living grapevine wood, or to other biotic and abiotic factors might also be relevant to explain the growing importance of Fmed in vineyards.

## Data availability statement

The mass spectrometry proteomics data have been deposited to the ProteomeXchange Consortium via the PRIDE (Perez-Riverol et al., 2022) partner repository with the dataset identifier PXD036889.

## Author contributions

EG, SF, CB, and MS contributed to conception and design of the study. MS (all data), ER (wood fibers data), AM-G, and RB (metabolomic data) contributed to obtain the experimental data. MS and EG (all data), ER (wood fibers data), AM-G, and RB and PH (metabolomic analysis) contributed to data analyses. MS wrote the first draft of the manuscript. All authors contributed to the article and approved the submitted version.

## Funding

UMR IAM is supported by the French Agency through the Laboratory of Excellence Arbre (ANR-11-LABX-0002-01), the region Grand Est and the Fonds Européen de Développement Régional (project VitEST). This work benefited from the ASIA platform (Université de Lorraine-INRAE; https://a2f.univ-lorraine.fr/en/asia-2/). Proteomics analyses were performed on the PAPPSO platform (http://pappso.inra.fr) which is supported by INRAE (http://www.inrae.fr), the Ile-de-France regional council (https://www.iledefrance.fr/education-recherche), IBiSA (https://www.ibisa.net) and CNRS (http://www.cnrs.fr).

## Conflict of interest

The authors declare that the research was conducted in the absence of any commercial or financial relationships that could be construed as a potential conflict of interest.

## Publisher’s note

All claims expressed in this article are solely those of the authors and do not necessarily represent those of their affiliated organizations, or those of the publisher, the editors and the reviewers. Any product that may be evaluated in this article, or claim that may be made by its manufacturer, is not guaranteed or endorsed by the publisher.

## Supplementary material

SUPPLEMENTARY FIGURE S1 Heatmap of global metabolite profiles during the wood degradation process. Log10 mean peak area of the indicated metabolites are given by shades of red, yellow, or blue colors according to the scale bar for **(A)** grapevine, **(B)** beech and **(C)** spruce cultures. Data represent the mean values of three biological replicates for each condition and time point. AA: amino acids; AL: aldehydes; F: flavonoid; PAc: phenolic acids; PAl: phenolic alcohols; St1–St4: Stilbene monomers, dimers, trimers and tetramers; MXXX: *m/z* in positive mode. C: control.

SUPPLEMENTARY FIGURE S2 Relative abundance expressed as emPAI for all selected glycoside hydrolases in **(A)** Fmed and **(B)** Tver secretome on grapevine and beech 1- and 3-month cultures. Proteins for which emPAI relative abundancy was higher than ten in at least one of the culture conditions were selected. RA: relative abundancy.

SUPPLEMENTARY TABLE S1 Relative abundancies expressed as emPAI for selected proteins in Fmed and Tver secretome on grapevine and beech cultures. The grouping of proteins into protein subgroups was done using the grouping algorithm included in X!TandemPipeline (Langella et al., 2017). Only proteins for which emPAI relative abundancy was higher than ten in at least one of the culture conditions were selected. Bulks are mix from all samples and were used as quality control during batch analysis. Protein IDs, annotations and classification are given according to JGI and Gene Ontology (GO) databases.
