# Supplementary figures and images for: Wood degradation by Fomitiporia mediterranea M. Fischer: Physiologic, metabolomic and proteomic approaches
Source: Front Plant Sci. 2022 Sep 26;13:988709. doi: 10.3389/fpls.2022.988709 (PMC9549746; doi:10.3389/fpls.2022.988709)

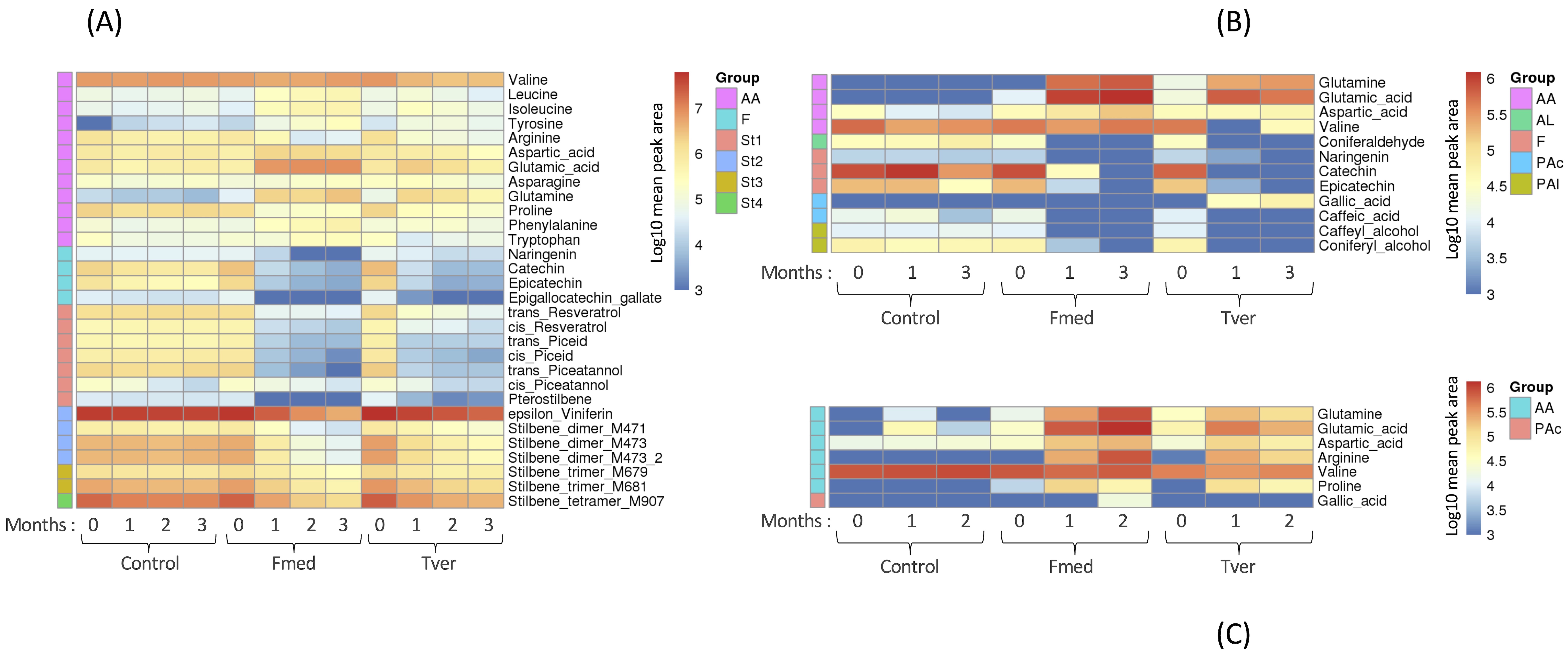

Supplement: SUPPLEMENTARY FIGURE S1 — Heatmap of global metabolite profiles during the wood degradation process. Log10 mean peak area of the indicated metabolites are given by shades of red, yellow, or blue colors according to the scale bar for (A) grapevine, (B) beech and (C) spruce cultures. Data represent the mean values of three biological replicates for each condition and time point. AA: amino acids; AL: aldehydes; F: flavonoid; PAc: phenolic acids; PAl: phenolic alcohols; St1–St4: Stilbene monomers, dimers, trimers and tetramers; MXXX: m/z in positive mode. C: control. [file Image_1.JPEG]

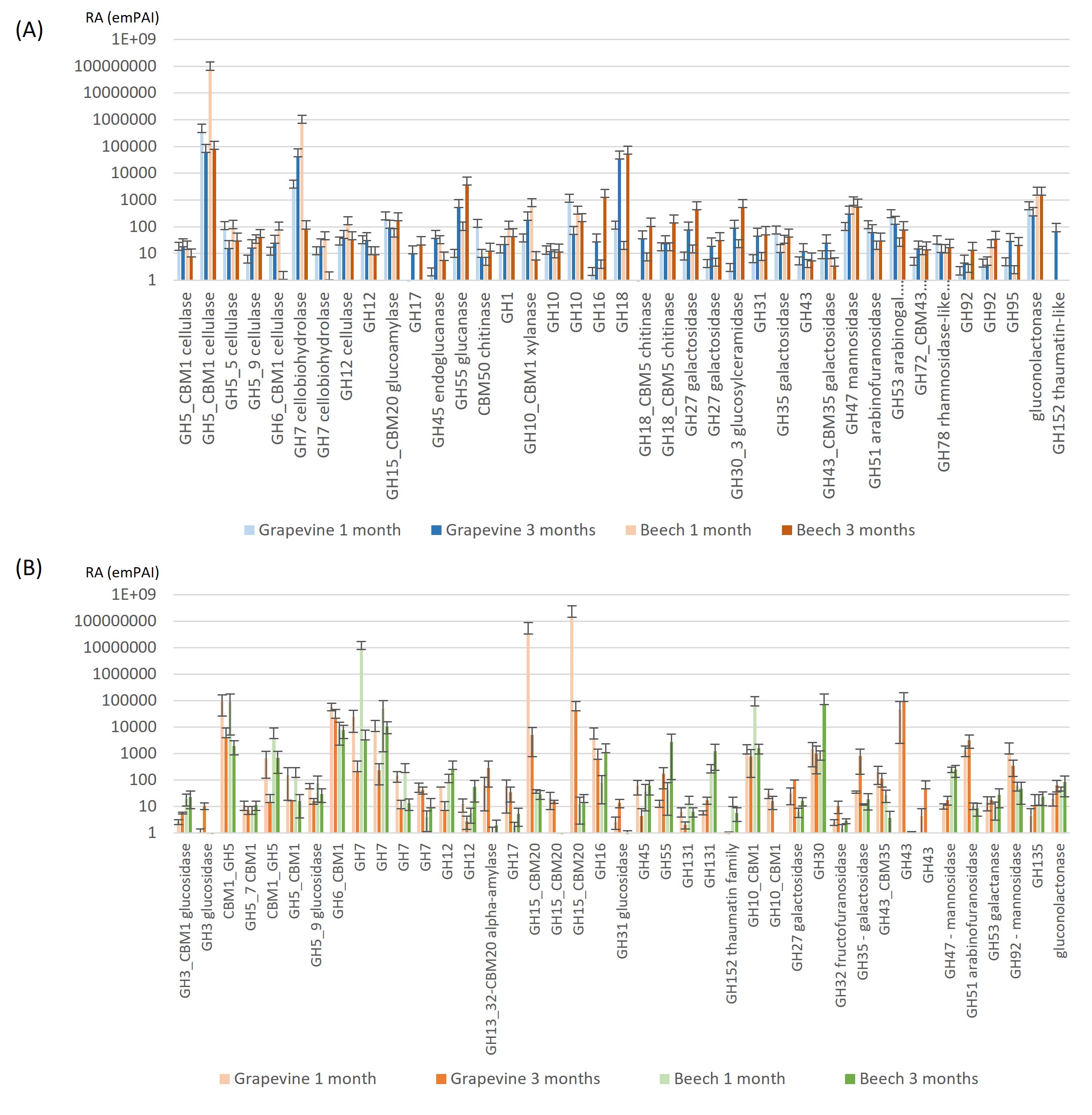

Supplement: SUPPLEMENTARY FIGURE S2 — Relative abundance expressed as emPAI for all selected glycoside hydrolases in (A) Fmed and (B) Tver secretome on grapevine and beech 1- and 3-month cultures. Proteins for which emPAI relative abundancy was higher than ten in at least one of the culture conditions were selected. RA: relative abundancy. [file Image_2.JPEG]
