# Supplementary material for: Wood degradation by Fomitiporia mediterranea M. Fischer: Physiologic, metabolomic and proteomic approaches
Source: Front Plant Sci. 2022 Sep 26;13:988709. doi: 10.3389/fpls.2022.988709 (PMC9549746; doi:10.3389/fpls.2022.988709)
Supplement: Supplementary file 4 [file Table_2.DOCX]

**Supplementary Table S2.** Number of CAZYs upon their classification in Fmed and Tver secretomes for each modality, according to JGI database, and after selection of proteins for which emPAI relative abundancy was higher than 10 in at least one of the culture conditions. ^1^Values for Fmed and Tver genomes are are given according to (Floudas et al., 2012). CAZYs: carbohydrate active enzymes, GH: glycoside hydrolase, CBM: cellulose binding module, AA9: auxiliary enzyme family 9 (syn. LPMO: lytic polysaccharide monooxygenase), CE: carbohydrate enzyme, PL: polysaccharides lyase

| CAZYs families | **Fmed** | | | | | | | | **Tver** | | | | | | | |
| --- | --- | --- | --- | --- | --- | --- | --- | --- | --- | --- | --- | --- | --- | --- | --- | --- |
|  | **Genome^1^** | All | Grapevine | | | Beech | | | G**enome^1^** | All | Grapevine | | | Beech | | |
|  |  |  | All | 1month | 3months | All | 1month | 3months |  |  | All | 1month | 3months | All | 1month | 3months |
| GH3 | **7** | 0 | 0 | 0 | 0 | 0 | 0 | 0 | **11** | 2 | 0 | 0 | 1 | 1 | 1 | 1 |
| GH5 | **6** | 4 | 4 | 3 | 4 | 4 | 4 | 3 | **5** | 5 | 4 | 5 | 4 | 3 | 4 | 5 |
| GH6 | **2** | 1 | 1 | 0 | 1 | 1 | 1 | 0 | **1** | 1 | 1 | 1 | 1 | 1 | 1 | 1 |
| GH7 | **2** | 2 | 2 | 1 | 2 | 2 | 2 | 1 | **4** | 4 | 4 | 3 | 3 | 3 | 3 | 3 |
| GH12 | **3** | 2 | 2 | 2 | 2 | 1 | 1 | 1 | **5** | 2 | 1 | 2 | 0 | 1 | 1 | 2 |
| GH13 | **-** | 0 | 0 | 0 | 0 | 0 | 0 | 0 | **-** | 1 | 1 | 1 | 1 | 1 | 0 | 0 |
| GH15 | **-** | 1 | 1 | 1 | 1 | 1 | 1 | 1 | **-** | 3 | 3 | 3 | 3 | 2 | 2 | 2 |
| GH17 | **-** | 1 | 0 | 0 | 0 | 0 | 0 | 1 | **-** | 1 | 1 | 1 | 1 | 1 | 0 | 0 |
| GH45 | **-** | 1 | 1 | 0 | 1 | 1 | 1 | 0 | **-** | 1 | 1 | 1 | 0 | 1 | 1 | 1 |
| GH55 | **-** | 1 | 1 | 0 | 1 | 1 | 1 | 1 | **-** | 1 | 1 | 1 | 1 | 1 | 1 | 1 |
| GH131 | **-** | 0 | 0 | 0 | 0 | 0 | 0 | 0 | **-** | 2 | 1 | 0 | 1 | 1 | 2 | 1 |
| GH with CBM50 | **-** | 1 | 1 | 1 | 0 | 0 | 0 | 1 | **-** | 0 | 0 | 0 | 0 | 0 | 0 | 0 |
| AA9 | **13** | 2 | 2 | 2 | 2 | 2 | 2 | 2 | **18** | 5 | 3 | 4 | 1 | 3 | 3 | 3 |
| GH1 | **-** | 1 | 1 | 1 | 1 | 1 | 1 | 1 | **-** | 0 | 0 | 0 | 0 | 0 | 0 | 0 |
| GH10 | **4** | 3 | 3 | 2 | 3 | 2 | 2 | 2 | **6** | 2 | 2 | 2 | 2 | 1 | 1 | 1 |
| GH16 | **-** | 1 | 1 | 0 | 1 | 1 | 0 | 1 | **-** | 1 | 1 | 1 | 1 | 1 | 1 | 1 |
| GH18 | **-** | 3 | 3 | 2 | 3 | 3 | 2 | 3 | **-** | 0 | 0 | 0 | 0 | 0 | 0 | 0 |
| GH27 | **-** | 2 | 2 | 0 | 2 | 2 | 1 | 2 | **-** | 2 | 2 | 2 | 2 | 2 | 1 | 2 |
| GH28 | **16** | 3 | 2 | 2 | 2 | 2 | 2 | 2 | **11** | 3 | 2 | 2 | 2 | 2 | 2 | 2 |
| GH30 | **-** | 1 | 1 | 0 | 1 | 1 | 1 | 1 | **-** | 1 | 1 | 1 | 1 | 1 | 1 | 1 |
| GH31 | **-** | 1 | 1 | 0 | 1 | 1 | 0 | 1 | **-** | 1 | 0 | 0 | 1 | 0 | 0 | 0 |
| GH32 | **-** | 0 | 0 | 0 | 0 | 0 | 0 | 0 | **-** | 1 | 0 | 0 | 1 | 0 | 0 | 0 |
| GH35 | **-** | 1 | 1 | 1 | 1 | 1 | 1 | 1 | **-** | 1 | 1 | 1 | 1 | 1 | 1 | 1 |
| GH43 | **6** | 2 | 1 | 0 | 2 | 0 | 0 | 0 | **3** | 3 | 3 | 2 | 3 | 1 | 1 | 0 |
| GH47 | **-** | 1 | 1 | 1 | 1 | 1 | 1 | 1 | **-** | 1 | 1 | 1 | 1 | 1 | 1 | 1 |
| GH51 | **-** | 1 | 1 | 1 | 1 | 1 | 1 | 1 | **-** | 1 | 1 | 1 | 1 | 0 | 1 | 0 |
| GH53 | **-** | 1 | 1 | 1 | 1 | 1 | 1 | 1 | **-** | 1 | 1 | 1 | 1 | 1 | 0 | 1 |
| GH72 | **-** | 1 | 0 | 0 | 1 | 1 | 0 | 1 | **-** | 0 | 0 | 0 | 0 | 0 | 0 | 0 |
| GH74 | **4** | 0 | 0 | 0 | 0 | 0 | 0 | 0 | **1** | 0 | 0 | 0 | 0 | 0 | 0 | 0 |
| GH78 | **-** | 1 | 1 | 1 | 1 | 1 | 1 | 1 | **-** | 0 | 0 | 0 | 0 | 0 | 0 | 0 |
| GH79 | **-** | 1 | 1 | 1 | 1 | 0 | 0 | 1 | **-** | 0 | 0 | 0 | 0 | 0 | 0 | 0 |
| GH92 | **-** | 2 | 0 | 0 | 0 | 1 | 1 | 2 | **-** | 1 | 1 | 1 | 1 | 1 | 1 | 1 |
| GH95 | **-** | 1 | 1 | 0 | 1 | 0 | 0 | 1 | **-** | 0 | 0 | 0 | 0 | 0 | 0 | 0 |
| GH135 | **-** | 0 | 0 | 0 | 0 | 0 | 0 | 0 | **-** | 1 | 0 | 0 | 1 | 1 | 1 | 1 |
| GH152 | **-** | 1 | 1 | 0 | 1 | 0 | 0 | 0 | **-** | 1 | 0 | 0 | 0 | 0 | 1 | 0 |
| Non-classified | **-** | 1 | 1 | 1 | 1 | 1 | 1 | 1 | **-** | 1 | 1 | 1 | 1 | 1 | 1 | 1 |
| CE1 | **0** | 1 | 1 | 1 | 1 | 1 | 1 | 1 | **3** | 2 | 2 | 2 | 2 | 2 | 2 | 2 |
| CE4 | **-** | 2 | 2 | 0 | 2 | 2 | 2 | 2 | **-** | 1 | 0 | 0 | 0 | 1 | 0 | 1 |
| CE7 | **-** | 2 | 1 | 0 | 1 | 2 | 2 | 2 | **-** | 0 | 0 | 0 | 0 | 0 | 0 | 0 |
| CE8 | **3** | 1 | 0 | 1 | 0 | 0 | 0 | 1 | **2** | 1 | 1 | 1 | 1 | 1 | 1 | 1 |
| CE12 | **2** | 0 | 0 | 0 | 0 | 0 | 0 | 0 | **0** | 0 | 0 | 0 | 0 | 0 | 0 | 0 |
| CE15 | **1** | 1 | 1 | 1 | 1 | 1 | 1 | 1 | **2** | 1 | 1 | 1 | 1 | 1 | 1 | 1 |
| CE16 | **6** | 1 | 1 | 0 | 1 | 1 | 1 | 1 | **7** | 3 | 2 | 1 | 2 | 2 | 1 | 3 |
| PL1 | **-** | 1 | 1 | 1 | 1 | 1 | 1 | 0 | **-** | 0 | 0 | 0 | 0 | 0 | 0 | 0 |
| PL8 | **-** | 0 | 0 | 0 | 0 | 0 | 0 | 0 | **-** | 1 | 1 | 0 | 1 | 0 | 0 | 0 |
